# Different phenotypes and chondrogenic responses of human menstrual blood and bone marrow mesenchymal stem cells to activin A and TGF-β3

**DOI:** 10.1186/s13287-021-02286-w

**Published:** 2021-04-29

**Authors:** Ilona Uzieliene, Edvardas Bagdonas, Kazuto Hoshi, Tomoaki Sakamoto, Atsuhiko Hikita, Zivile Tachtamisevaite, Greta Rakauskiene, Giedrius Kvederas, Ali Mobasheri, Eiva Bernotiene

**Affiliations:** 1 Department of Regenerative Medicine, State Research Institute Centre for Innovative Medicine, LT-08406 Vilnius, Lithuania; 2 Department of Sensory and Motor System Medicine, Department of Oral-maxillofacial Surgery, Dentistry and Orthodontics, Graduate School of Medicine, The University of Tokyo, Hongo 7-3-1, Bunkyo-ku, Tokyo, 113-8655 Japan; 3 Department of Tissue Engineering, the University of Tokyo Hospital, Hongo 7-3-1, Bunkyo-ku, Tokyo, 113-8655 Japan; 4 Faculty of Medicine, Vilnius University, Vilnius, Lithuania; 5 Research Unit of Medical Imaging, Physics and Technology, Faculty of Medicine, University of Oulu, FI-90014 Oulu, Finland; 6 Departments of Orthopedics, Rheumatology and Clinical Immunology, University Medical Center Utrecht, 508 GA Utrecht, The Netherlands; 7 Department of Joint Surgery, First Affiliated Hospital of Sun Yat-sen University, Guangzhou, Guangdong China

**Keywords:** Human mesenchymal stem cells, Menstrual blood, Bone marrow, Chondrogenic differentiation, Activin A, TGF-β3

## Abstract

**Background:**

Due to its low capacity for self-repair, articular cartilage is highly susceptible to damage and deterioration, which leads to the development of degenerative joint diseases such as osteoarthritis (OA). Menstrual blood-derived mesenchymal stem/stromal cells (MenSCs) are much less characterized, as compared to bone marrow mesenchymal stem/stromal cells (BMMSCs). However, MenSCs seem an attractive alternative to classical BMMSCs due to ease of access and broader differentiation capacity. The aim of this study was to evaluate chondrogenic differentiation potential of MenSCs and BMMSCs stimulated with transforming growth factor β (TGF-β3) and activin A.

**Methods:**

MenSCs (*n* = 6) and BMMSCs (*n* = 5) were isolated from different healthy donors. Expression of cell surface markers CD90, CD73, CD105, CD44, CD45, CD14, CD36, CD55, CD54, CD63, CD106, CD34, CD10, and Notch1 was analyzed by flow cytometry. Cell proliferation capacity was determined using CCK-8 proliferation kit and cell migration ability was evaluated by scratch assay. Adipogenic differentiation capacity was evaluated according to Oil-Red staining and osteogenic differentiation according to Alizarin Red staining. Chondrogenic differentiation (activin A and TGF-β3 stimulation) was investigated in vitro and in vivo (subcutaneous scaffolds in nude BALB/c mice) by expression of chondrogenic genes (collagen type II, aggrecan), GAG assay and histologically. Activin A protein production was evaluated by ELISA during chondrogenic differentiation in monolayer culture.

**Results:**

MenSCs exhibited a higher proliferation rate, as compared to BMMSCs, and a different expression profile of several cell surface markers. Activin A stimulated collagen type II gene expression and glycosaminoglycan synthesis in TGF-β3 treated MenSCs but not in BMMSCs, both in vitro and in vivo, although the effects of TGF-β3 alone were more pronounced in BMMSCs in vitro.

**Conclusion:**

These data suggest that activin A exerts differential effects on the induction of chondrogenic differentiation in MenSCs vs. BMMSCs, which implies that different mechanisms of chondrogenic regulation are activated in these cells. Following further optimization of differentiation protocols and the choice of growth factors, potentially including activin A, MenSCs may turn out to be a promising population of stem cells for the development of cell-based therapies with the capacity to stimulate cartilage repair and regeneration in OA and related osteoarticular disorders.

## Introduction

Human articular cartilage has a poor capacity for intrinsic repair and a weak ability to restore its lesions leading to the development of progressive and degenerative diseases such as osteoarthritis (OA), which is one of the most common forms of arthritis across the world [1]. Currently, there is no effective treatment for OA, although cell-based therapies using mesenchymal stem/stromal cells (MSCs) seem promising approaches for cartilage regeneration. However, thus far, the potential to effectively restore damaged cartilage in OA, using MSC-based therapies, remain challenging [2–4]. Furthermore, due to complications associated with the collection of bone marrow (BM) samples, which is an invasive procedure, and the poor yield of MSCs, alternative sources of cells are needed in order to overcome technical issues in BMMSC isolation, for instance from, adipose, amniotic, and umbilical cord tissues [2, 5, 6]. Menstrual blood is a unique body fluid, a renewable and sustainable source of multipotent stem cells (MenSCs) for regenerative medicine, which may originate from different subpopulations of endometrium, as reviewed [7, 8]. These cells are of great interest due to their ease of access, as their collection does not require any complicated procedures, permission of ethics authorities, or invasive surgical procedures [9, 10]. Noteworthy, some studies report no difference in male vs. female MSCs [11], while others suggest that female BMMSCs offer more advantages [12]. Moreover, previous studies have demonstrated that MenSCs can even be differentiated towards a wider range of neurogenic, cardiomyogenic, and hepatogenic lineages [13–16]. Chondrogenic differentiation of MenSCs has been reported in several studies, suggesting that MenSCs could be a suitable candidate for cartilage tissue engineering as they have been reported to produce and accumulate a cartilage-specific extracellular matrix (ECM) [16, 17]. However, the expression of collagen 2A1 mRNA was exclusively detectable in differentiated BMMSCs, whereas it was not observed in differentiated MenSCs [14], suggesting that molecular mechanisms regulating chondrogenic differentiation of MenSCs may differ in these two cells and the classical protocol applied for BMMSCs might not be suitable for MSCs of other tissue origins [18, 19]. Classical chondrogenic stimulating factors, such as TGF-β and bone morphogenetic protein-2 (BMP-2), may not stimulate differentiation in MenSCs [20–22] and application of alternative stimulating factors may be needed.

Several studies suggest that activin A, a member of the TGF-β superfamily, could play a role in the early stages of MSC chondrogenesis [23, 24]. Activin A induces the expression of octamer-binding transcription factor 4 (Oct4), nanog, nodal, proto-oncogene protein Wnt3, and fibroblast growth factor 8 (FGF-8) and is necessary for the maintenance of self-renewal and pluripotency of MSCs [23]. Furthermore, enhanced production of activin A was demonstrated in OA cartilage, as compared to the healthy one, where it suppressed aggrecanase-mediated cleavage of aggrecan [25]. This suggests activin A to be a protective factor for OA development, and a potential candidate for future cartilage repair strategies.

The aim of this study was to evaluate the chondrogenic differentiation potential of MenSCs and BMMSCs stimulated with TGF-β3 and/or activin A in cell pellets and on atelocollagen/polylactic acid (PLLA) scaffolds, subcutaneously inserted into nude BALB/c mice for 9 weeks. We also compared the phenotypic properties of MenSCs and BMMSCs by characterizing the expression of cell surface cluster of differentiation (CD) markers and their proliferation capacity and potential to differentiate into adipogenic and osteogenic MSC lineages.

## Materials and methods

### Cell isolation and culture

Menstrual blood samples were collected from six healthy women aged between 20 and 40. About 5–10 mL of menstrual blood was collected by donors using sterile silicone cups (iCare) inserted into the vagina during second day of menstrual cycle. Mononuclear cells were separated using Ficoll-Paque PLUS (Stem Cell Technologies) density gradient centrifugation (30 min, room temperature, 400 *g*) and washed out two times in phosphate-buffered saline (PBS) (Sigma Aldrich), centrifuged 10 min, 600 *g*. Collected cells were seeded into tissue culture flasks (Gibco, Life Technologies) with low glucose (1 g/L) Dulbecco’s modified Eagle medium (DMEM) (Merck Millipore) supplemented with 10% fetal bovine serum (FBS) (Merck Millipore), 1% penicillin/streptomycin (Gibco, Life Technologies), 1 ng/mL FGF2 (Applied Biological Materials) (later referred as “complete medium”), and cultured in 37 °C incubator with 5% CO_2_, saturated humidity. Medium was changed twice a week, and after cells reached their confluence (~ 80%), they were detached using trypsin-EDTA 0.25% solution (Gibco, Life Technologies), counted (CASY, Omni Life Science), and sub-cultured. Human bone marrow cells were isolated from bone marrow tissues, remaining after surgical procedures according to the established protocols as previously described [26]. Briefly, bone marrow tissues were excised from bone using a scalpel and collected into a sterile tube. The collected tissue samples and cells were diluted with sterile PBS with 1% penicillin/streptomycin, centrifuged 10 min, 450 *g*, and filtered through 100 μm filter. Then, the bone marrows mononuclear cells were seeded into tissue culture flasks. There were five bone-marrow donors (3 female, 2 male) aged between 50 and 60. Bone marrow mononuclear cells were cultured under the same conditions as menstrual blood-derived cells. All procedures using human tissues in this study were approved by the local bioethics committee, permission no. 158200-14-741.

The experiments were performed using menstrual blood and bone marrow isolated cells at early passages (P) P2 to P3.

### Immunophenotypic characterization of cells

CD surface marker analysis was performed by harvesting the cells, washing them with cytometer buffer (PBS + 2% bovine serum albumin (BSA) (Biological Industries)) 5 min at 600 *g* and incubating with the specific labeled antibodies in cytometer buffer for 20 min at 4 °C. Primary antibodies with fluorophores were used for the experiment against human cell surface cluster of differentiation CD antigens CD90, CD73, CD105, CD44, CD45, CD14, CD36, CD55, CD54, CD63, CD34, CD10, and Notch1 (see Table 1).

**Table 1:** Antibodies used in this study and their sources

Name	Protein	Fluorophore	Isotype	Source
Anti–CD90	Glycoprotein	FITC	IgG1	Bio Legend
Anti–CD73	5′-Nucleotidase	PE	IgG1	Bio Legend
Anti–CD105	Glycoprotein	APC	IgG1	BD Biosciences
Anti–CD44	Glycoprotein	FITC	IgG2b	BD Biosciences
Anti–CD45	Receptor	FITC	IgG2b	Santa Cruz Biotechnology
Anti–CD14	Receptor	APC	IgG2a	Bio Legend
Anti–CD36	Glycoprotein	APC	IgM	BD Biosciences
Anti–CD55	Glycoprotein	PE	IgG2a	Santa Cruz Biotechnology
Anti–CD54	Adhesion molecule	APC	IgG1	BD Biosciences
Anti–CD63	Tetraspanin	PE	IgG1	BD Biosciences
Anti–CD34	Glycoprotein	PE	IgG1	Bio Legend
Anti–CD10	Endopeptidase Metalloproteinase	FITC	IgG1	Bio Legend
Anti–Notch1	Receptor	APC	IgG1	eBioscience
IgG1	Isotype control	PE	IgG1	Santa Cruz Biotechnology
IgG1	Isotype control	FITC	IgG1	Santa Cruz Biotechnology
IgG1	Isotype control	APC	IgG1	Santa Cruz Biotechnology
IgG2a	Isotype control	APC	IgG2a	Bio Legend
IgG2a	Isotype control	PE	IgG2a	Bio Legend
IgG2b	Isotype control	FITC	IgG2b	Bio Legend
IgM	Isotype control	APC	IgM	BD Biosciences

Each marker was assessed separately for each sample. In all experiments, matching isotype antibodies were used as negative controls. One technical replicate was used for each donor. 7AAD dye was used for viability staining. Data (10.000 events) were collected using a flow cytometer FACS Aria II (BD Biosciences) and analyzed on FacsDiva analysis software (BD Biosciences). The data was analyzed excluding non-viable cells and gating out isotype controls.

### Adipogenic and osteogenic differentiation

For adipogenic differentiation, MenSCs and BMMSCs were seeded into a 12-well plate, at a density of 60,000 cells/well. Differentiation was induced in sub-confluent cells using adipogenic medium, consisting of low glucose (1 g/L) DMEM medium, 1% penicillin/streptomycin, 20% FBS, 1 μmol/L dexamethasone (Sigma Aldrich), 60 μmol/L indomethacin (Sigma Aldrich), and 50 μmol/L 3-isobutyl-1-methylxanthine (IBMX) (Biosource) for 21 days. After that, lipid droplets in cells were stained with Oil Red-O (Carl Roth) and visualized in an inverted light microscope. Staining procedures were used as discussed in our previous paper [27]. Quantitative analysis was performed by dissolving lipid droplets in 70% isopropanol solution (Eurochemicals), after which the released oil-red dye was collected into a 96-well plate and measured absorbance using spectrophotometer (absorbance, 520 nm).

For osteogenic differentiation, MenSCs and BMMSCs were also seeded into a 12-well plate, at a density 40,000 cells/well. Osteogenic differentiation medium was added after the cells reached their sub-confluence, consisting of high glucose (4.5 g/L) DMEM medium (Gibco, Life Technologies), 1% penicillin/streptomycin, 10% FBS, 0.1 μmol/L dexamethasone, 50 μg/mL ascorbic acid, and 10 mmol/L β – glycerophosphate (Santa Cruz) for 21 days. Osteogenesis was evaluated by light microscopy, staining the cells with Alizarin Red S (Carl Roth). Quantitative analysis was evaluated by dissolving the calcium hydroxyapatite crystals, stained with Alizarin, in 10% cetylpyridinium chloride (Sigma Aldrich). The dissolved solution was then collected into 96-well plate and absorbance measured using spectrophotometer (absorbance, 562 nm).

Three technical replicates were used for each donor and undifferentiated cells were used as controls for immunostaining. Data was presented as ratio to controls.

### Proliferation assay

MenSCs and BMMSCs were seeded into 12-well plates (SPL, Life Sciences) at density 5000 cells/cm^2^ in a complete medium. Cell proliferation was determined at days 1, 5, 8, and 12 by measuring it with cell counting kit – 8 (CCK-8) (Dojindo) according to manufacturer’s instructions. Commercial CCK-8 kit allows to measure cell proliferation and cytotoxicity at once, by utilizing water-soluble tetrazolium salt. This salt is reduced by dehydrogenases of living cells and produces an orange-colored formazan dye. The amount of formazan dye generated by cell dehydrogenases is directly proportional to the number of living cells. Three technical replicates were used for each donor. Complete medium was used as blank. The medium was collected to 96-well plate (Orange Scientific), and absorbance of reduced formazan dye was quantified using spectrophotometric quantification (SpectroMaxi3, Molecular Devices) (absorbance, 450 nm). For control, complete medium was used with the same amount of CCK-8 reagent.

### Scratch assay

Scratch assay was performed to evaluate cell migration capacity. MenSCs and BMMSCs were seeded into T25 flasks (Gibco, Life Technologies) and grown to full confluence. Later, a straight scratch of the cell monolayer was performed with a 1 mL pipet tip. The cell scratch was photographed under a phase-contrast microscope at 0, 8, 24, and 48 h, and cell migration abilities were evaluated by the size of area of migrated cells inside the scratch was evaluated visually by using the same magnification in the same location. Three technical replicates were used for each donor.

### Chondrogenic differentiation using activin A and TGF-β3 in vitro

Chondrogenesis was induced using a protocol used by State Research Institute Centre for Innovative medicine. Chondrogenic medium was composed of high glucose (4.5 g/L) DMEM medium, 1% penicillin/streptomycin, 1% insulin-transferrin-selenium (Gibco Life Technologies), 350 μM L-proline (Carl Roth), 0.1% dexamethasone, 170 μM ascorbic acid-phosphate (Sigma Aldrich) and 10 ng/mL TGF-β3 (Gibco, Life Technologies), or 50 ng/mL activin A (Merck, Millipore). Briefly, 250k of MenSCs or BMMSCs were added in 15 mL tubes (Gibco, Life Technologies), centrifuged 5 min at 600 *g*, and cultivated in chondrogenic medium. The tubes with cells were divided into 4 groups: (1) control (chondrogenic medium without growth factors), (2) activin A, (3) TGF-β3, and (4) activin A + TGF-β3. Double growth factor group was stimulated with both factors only once at the start of differentiation, while during the second medium change on day 2, only TGF-β3 was added. TGF-β3 was added throughout the entire process. This protocol was chosen based on previous studies suggesting the efficacy of activin A at the early stages of chondrogenic differentiation [23]. Each treatment was applied in technical triplicates. The production of ECM in cell pellets was evaluated macroscopically and histologically (Safranin O staining). The amount of stained proteoglycans was visually compared to control samples, which were not stimulated with any of the growth factors.

### RNA extraction from cell pellets

At the end of chondrogenic differentiation period, the cell pellets were collected, flash-frozen in liquid nitrogen, and stored at − 70 °C. Frozen samples were homogenized by ultrasonication (Bandelin Sonopuls) in Qiazol lysis buffer (Qiagen), and RNA was extracted according manufacturer’s protocol. RNA concentration and purity were measured with the SpectraMax i3 (Molecular Devices, USA).

### RT-qPCR

RNA was reverse-transcribed with Maxima cDNA Synthesis kit including dsDNase treatment (Thermo Scientific). RT-qPCR reaction mixes were prepared with Maxima Probe qPCR Master Mix (Thermo Scientific) and TaqMan Gene expression Assays (*RPS9* - Hs02339424_g1, *B2M* - Hs00984230_m1, *COL2A1* - Hs01060345_m1, *ACAN* - Hs00153936_m1 (Thermo Scientific)) and ran on the Agilent Aria MX instrument (Agilent Technologies) in technical triplicates starting with denaturation step for 10 min at 95 °C followed by 40 cycles of 15 s at 95 °C denaturation and 60 s for annealing and extension. Relative levels of gene transcripts were calculated by subtracting the threshold cycle (Ct) of the normalizer (the geometric mean of the two housekeeping genes—RPS9 and B2M) from the Ct of the gene of interest, giving the dCt values which were subsequently transformed to 2^−dCt^ values and multiplied by 1000 to scale-up for better graphical representation.

### Quantification of activin A from chondrogenic pellet supernatants and monolayer by ELISA

Activin A protein production was evaluated during chondrogenic differentiation of MenSCs and BMMSCs and during cell cultivation in monolayer. Supernatants were collected from the pellets incubated with TGF-β3 and control during chondrogenic differentiation and from monolayer cultures during 8th and 12th days (12 pm). Activin A stimulated samples were not assessed in this study. Activin A protein levels were detected using activin A duo set ELISA (R&D Systems) according to the manufacturer’s protocol. Activin A levels secreted during monolayer culture were normalized according to upgrowing number of cells by precise value of proliferation. It was implemented by calculating the ratio of a secreted activin A to proliferation value.

### Chondrogenic differentiation using activin A and TGF-β3 in vivo

Chondrogenic differentiation of MenSCs (3 donors) and BMMSCs (3 donors) was also stimulated in atelocollagen/PLLA constructs, which were inserted into six nude mice (BALB/c). For this purpose, 100,000 cells were mixed with 100 μL of atelocollagen gel (Koken) for 10 min according to the manufacturer’s recommendations, transferred to PLLA (R&D), and incubated for 2 h in 37 °C incubator with 5% CO_2_. After that, cell constructs were transferred into 20 mL tubes and chondrogenic differentiation medium was added. The constructs with cells of each donor were divided into three groups: (1) control, (2) TGF-β3, and (3) activin A + TGF-β3, where (1) control cells were incubated in chondrogenic medium without growth factors, (2) cells incubated in chondrogenic medium with TGF-β3 (10 ng/mL, 21 days), and (3) a combination of activin A (50 ng/mL), which was added only once, for 2 days at the beginning of the differentiation with TGF-β3 (10 ng/mL) for 21 days. The medium was changed three times a week. After the end of differentiation, the cell constructs were subcutaneously implanted into BALB/c nude mice (*n* = 6), under general anesthesia with isoflurane (Sigma Aldrich). Each donor’s cell constructs (control, activin A, and TGF-β3) were inserted into the same mouse. The constructs with cells remained in mice for 9 weeks. After that period, the mice were sacrificed using diethyl ether (Sigma Aldrich) and the constructs were carefully removed. Every construct was divided into two equal parts, where one part was used for histological analysis and the other for GAG protein analysis (Biocolor).

### GAG analysis in cell atelocollagen/PLLA constructs

Half of the collected cell atelocollagen/PLLA constructs were transferred into acetic acid solution (50 nM) (Sigma Aldrich) and homogenized using stainless steel beads in homogenizer (300 s, 3200 rpm). After homogenization, all procedures were performed on ice. The homogenized solution was transferred to 1.5 mL conical tubes and pepsin (10 ng/mL) (Wako) was added for 48 h, 4 °C in a rotor spin. After 48 h, the solution was neutralized using Tris-buffered saline (TBS) buffer, consisting of 1 M Tris, 2 M sodium chloride, and 50 mM calcium chloride solution. Elastase (1 mg/mL) (Wako) was added overnight, 4 °C in a rotor spin. The following morning, the samples were centrifuged at 910 *g*, 5 min, 4 °C. The protein levels were standardized using Lowry method. The GAG levels were measured spectrophotometrically (660 nm) using blyscan™-sulfated GAG assay (Biocolor), according to the manufacturer’s recommendations, as previously described [28]. The controls and blanks were provided in a commercial kit.

### Histology of chondrogenic differentiation for cell pellets and atelocollagen/PLLA constructs

For histochemical analysis, chondrogenic differentiation pellets and excised atelocollagen scaffolds were fixed in 10% neutral formalin and embedded into paraffin. Three-micrometer sections were deparaffinized and processed for standard staining with Safranin O (Sigma Aldrich). The samples were deparaffinized and hydrated to distilled water, and then stained with 1% of Safranin O solution for 10 min. After, they were rinsed with distilled water and stained with fast green (Sigma Aldrich) solution for 15 s. After, the samples were cleared with 96% ethyl alcohol and xylene (all from Sigma Aldrich), and the slides were mounted using mounting medium (Sigma Aldrich).

### Statistical analysis of studies

Student’s *t* test was used to calculate statistical difference among MenSCs and BMMSCs data in proliferation, immunophenotypic, GAG analysis, gene analysis, and ELISA assays. A *P* value of ≤ 0.05 was considered statistically significant.

## Results

### Surface marker expression in MenSCs and BMMSCs

**Fig. 1:**
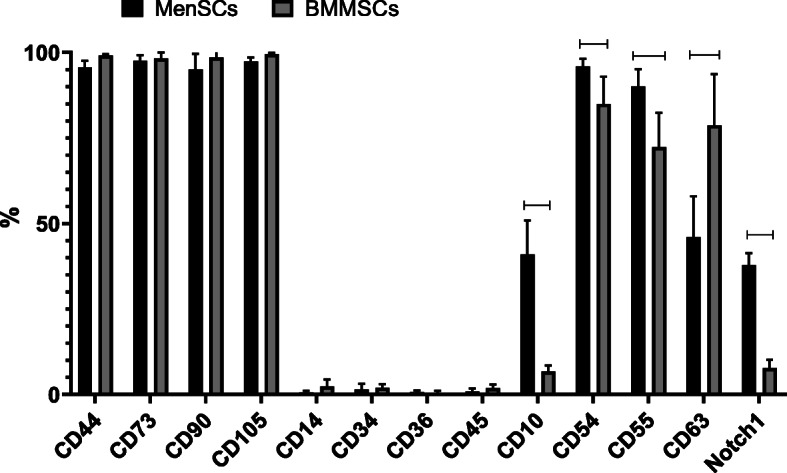
Expression of surface markers (CD44, CD73, CD90, CD105, CD14, CD34, CD36, CD45, CD10, CD54, CD55, CD63, Notch1) in MenSCs and BMMSCs. Flow cytometry analysis. %— percentage of cell population positive for indicated marker. Horizontal bars represent *p* ≤ 0.05

In order to determine and compare immunophenotypic profiles of MenSCs and BMMSCs, the cells were stained with antibodies against MSC surface markers (CD44, CD73, CD90, CD105), hematopoietic (CD14, CD34, CD36, CD45), and pluripotential stem cell markers (CD10, CD54, CD55, CD63, CD106, Notch1) and analyzed by flow cytometry (Fig. 1).

MenSCs and BMMSCs were both positive for classical MSC markers—CD44, CD73, CD90, and CD105—where more than 95% of total cell population was positive and negative for hematopoietic stem cell markers—CD14, CD34, CD36, and CD45 (less than 10% of total cell population was positive). Expression of CD55, CD54, CD10, and Notch1 was statistically significantly higher in MenSCs, as compared to BMMSCs, whereas CD63 was more expressed in BMMSCs. Average values—CD10: MenSCs 40.9 ± 0.12%; BMMSCs 6.7 ± 1.77%, *p* = 0.031; CD54: MenSCs 95.88 ± 2.2%; BMMSCs 84.93 ± 8%, *p* = 0.022; CD55: MenSCs 90.12 ± 5%; BMMSCs 72.35 ± 10%, *p* = 0.049; CD63: MenSCs 46.04 ± 11.9%; BMMSCs 78.70 ± 15%, *p* = 0.029; Notch1: MenSCs 37.84 ± 20%; BMMSCs 7.75 ± 2.4%, *p* = 0.035.

### MenSCs and BMMSCs ability to differentiate into osteoblasts and adipocytes

**Fig. 2:**
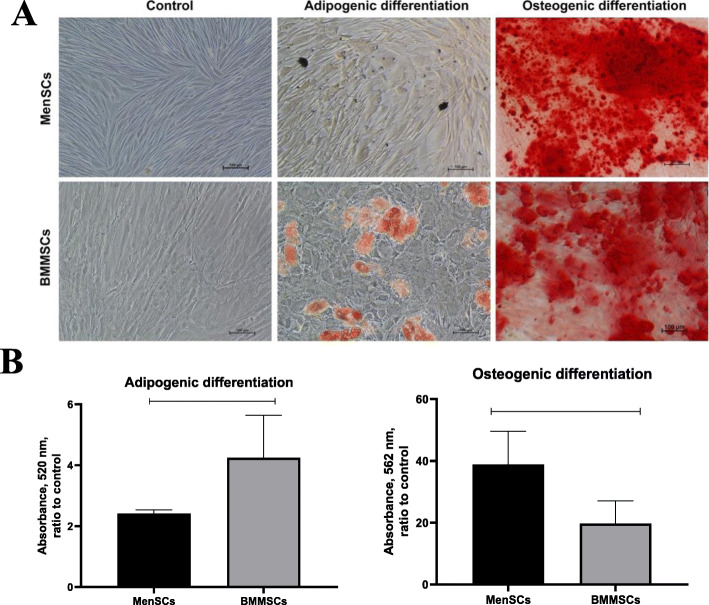
**a** Adipogenic and osteogenic differentiation analysis of MenSCs and BMMSCs, staining the cells with oil red, and alizarin, respectively. Control cells were incubated using standard complete DMEM medium and stained with the same dyes after differentiation ended. The cells were cultivated in adipogenic/osteogenic medium for 21 days. Scale bar 100 μm. **b** Quantitative adipogenic and osteogenic differentiation analysis of MenSCs and BMMSCs (21 days), dissolving lipid droplets (stained with oil-red) in isopropanol, and calcium hydroxyapatite crystals (stained with alizarin red) in cetylpyridinium chloride solutions. Absorbance measured at 520 nm and 562 nm, respectively. Data are presented as ratio to control, mean ± SD. Horizontal bars represent *p* ≤ 0.05

To evaluate mesodermal lineage potential, MenSCs and BMMSCs were induced to differentiate towards adipogenic and osteogenic direction (Fig. 2). The results demonstrate that MenSCs and BMMSCs are able to differentiate into adipogenic and osteogenic lineage cells. However, adipogenic differentiation capacity was significantly stronger in BMMSCs (3.22 ± 1.39 A.U.) than in MenSCs (2.41 ± 0.13 A.U.) (*p* = 0.012), as they formed more lipid droplets. However, osteogenic differentiation was significantly higher in MenSCs (38.86 ± 10.71 A.U.) than in BMMSCs (19.74 ± 7.32 A.U.) (*p* = 0.043).

### MenSC and BMMSC proliferation and migration capacity

**Fig. 3:**
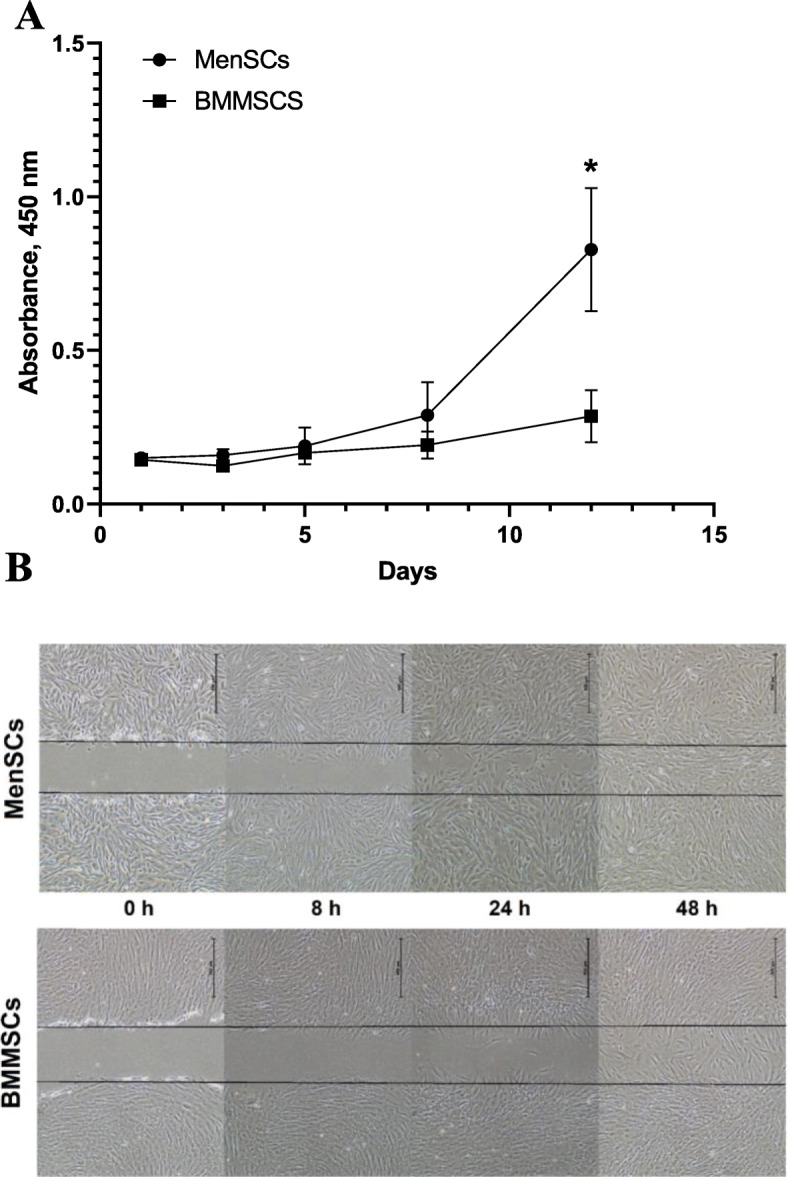
**a** Proliferation of MenSCs and BMMSCs in monolayer, during 1, 3, 5, 8, and 12 days of culture. Measured using CCK-8 viability and cytotoxicity assay. The absorbance of reduced formazan dye, produced by living cells, is presented. Absorbance measured at 450 nm. Data are presented as mean ± SD, *p ≤ 0.05. **b** Scratch assay. MenSCs and BMMSCs migration after mechanically disrupted monolayer with a sterile 1 ml pipet tip, obtained at different time points (0, 8, 24, and 48 h). Gap closure was assessed visually. Scale bar 500 μm

The proliferation capacity of MenSCs was significantly higher than BMMSCs (Fig. 3a). During the first 3 days of culture, there were no significant differences in cell proliferation; however, from the 5th day, MenSC proliferation started to increase, and on day 12, the difference reached statistical significance, as compared to BMMSCs (MenSCs 0.828 ± 0.201 A.U., BMMSCs 0.286 ± 0.085 A.U., *p* = 0.022).

In scratch assays, after 48 h, complete wound closure was observed in MenSCs, but not in BMMSCs (Fig. 3b). Gap closure was assessed visually.

These results suggest that proliferation and migration of MenSCs are higher than BMMSCs.

### Secretion of activin A during proliferation and chondrogenic differentiation of MenSCs and BMMSCs

**Fig. 4:**
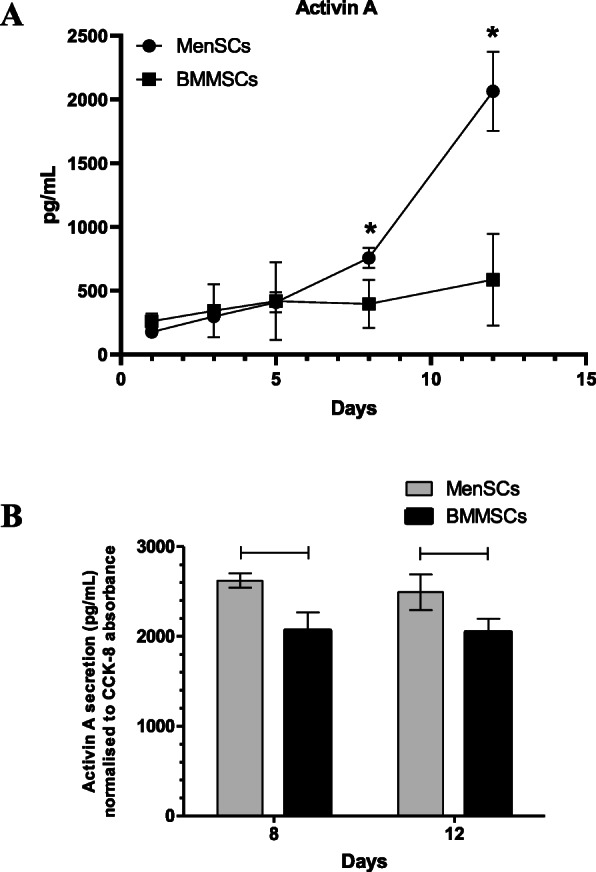
**a** Levels of activin A in supernatants of MenSCs and BMMSCs monolayer cultures on days 1, 3, 5, 8, and 12 of cell proliferation in complete DMEM medium. ELISA assay. Data presented as mean ± SD, *p ≤ 0.05. **b** Secreted levels of activin A, normalized according to CCK-8 absorbance of MenSCs and BMMSCs on days 8 and 12. Horizontal bars represent *p* ≤ 0.05

During 12 days of cell culture in monolayer (Fig. 4a), the level of activin A increased in supernatants, and during 8 and 12 days, it was significantly higher in MenSCs (757 ± 79 pg/mL, and 2064 ± 312 pg/mL, respectively), as compared to BMMSCs (396 ± 187.51 pg/mL and 586 ± 359.2 pg/mL, respectively). *P* values: 8th day—0.018; 12th day—0.028. We next wanted to determine whether the higher levels of activin A reflect the elevated secretion or rather the higher numbers of MenSCs due to the more pronounced proliferation. To investigate this, activin A secretion was normalized according to the upgrowing cell activity, measured by CCK-8, which essentially represents cell numbers (Fig. 4b). After normalization, secretion of activin A still remained significantly higher in MenSCs as compared to BMMSCs on days 8th and 12th in monolayer: (8th day—MenSCs 2622.8 ± 79.24; BMMSCs 2066.7 ± 200, *p* = 0.027; 12th day—MenSCs 2493.1 ± 200; BMMSCs 2046.7 ± 150, *p* = 0.031).

### Stimulation of chondrogenic differentiation in MenSCs and BMMSCs with activin A and/or TGF-β3

**Fig. 5:**
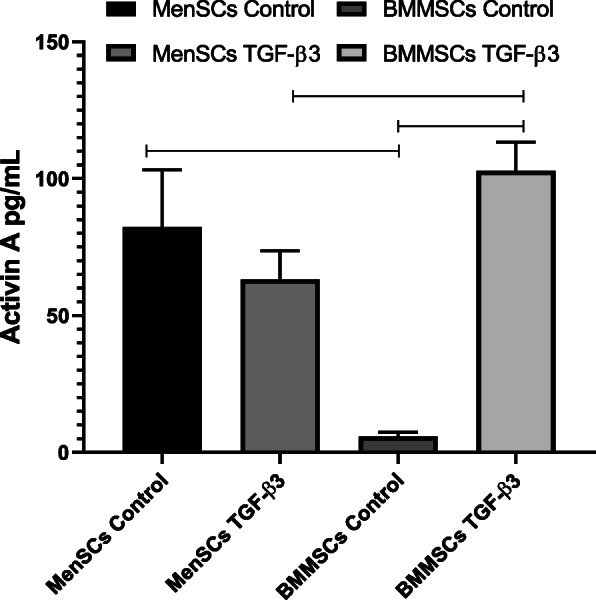
Levels of secreted activin A after 3 days of chondrogenic differentiation in MenSCs and BMMSCs in incomplete chondrogenic medium (control) or incomplete chondrogenic medium + TGF-β3 (10 ng/mL). Pellet cultures, ELISA assay. Data are presented as mean ± SD, horizontal bars represent *p* ≤ 0.05

After analysis of activin A secretion in monolayer, both cell types were stimulated to differentiate into the chondrogenic lineage using TGF-β3 and cell supernatants were collected for activin A secretion analysis. During the 3rd day of chondrogenic differentiation in the pellet system, the levels of activin A are presented in Fig. 5. In MenSCs, levels of activin A were significantly higher than in BMMSCs in both control cells (82.32 ± 2.94 pg/mL vs. 5.88 ± 2.94 pg/mL, respectively; *p* ≤ 0.001) and cells, stimulated with TGF-β3 on 3rd day of chondrogenic induction (63.21 ± 1.47 pg/mL vs. 102.9 ± 1.47 pg/mL, respectively; *p* ≤ 0.05 BMMSCs maintained production of activin A on 3rd day of differentiation only in combination with TGF-β3 (102.9 ± 1.47 pg/mL), as compared to control (5.88 ± 2.94 pg/mL) (*p* ≤ 0.05). Noteworthy, MenSCs control cells, which were incubated without any growth factors, produced higher quantities of activin A than cells stimulated with TGF-β3, where in contrast, BMMSCs maintained activin A secretion only under stimulation with TGF-β3. These differences might be associated with activin A-dependent mechanisms specific for MenSCs. During the following 19 days of chondrogenic induction of both MenSCs and BMMSCs, the secretion of activin A was very low and practically non-detectable (data not shown).

**Fig. 6:**
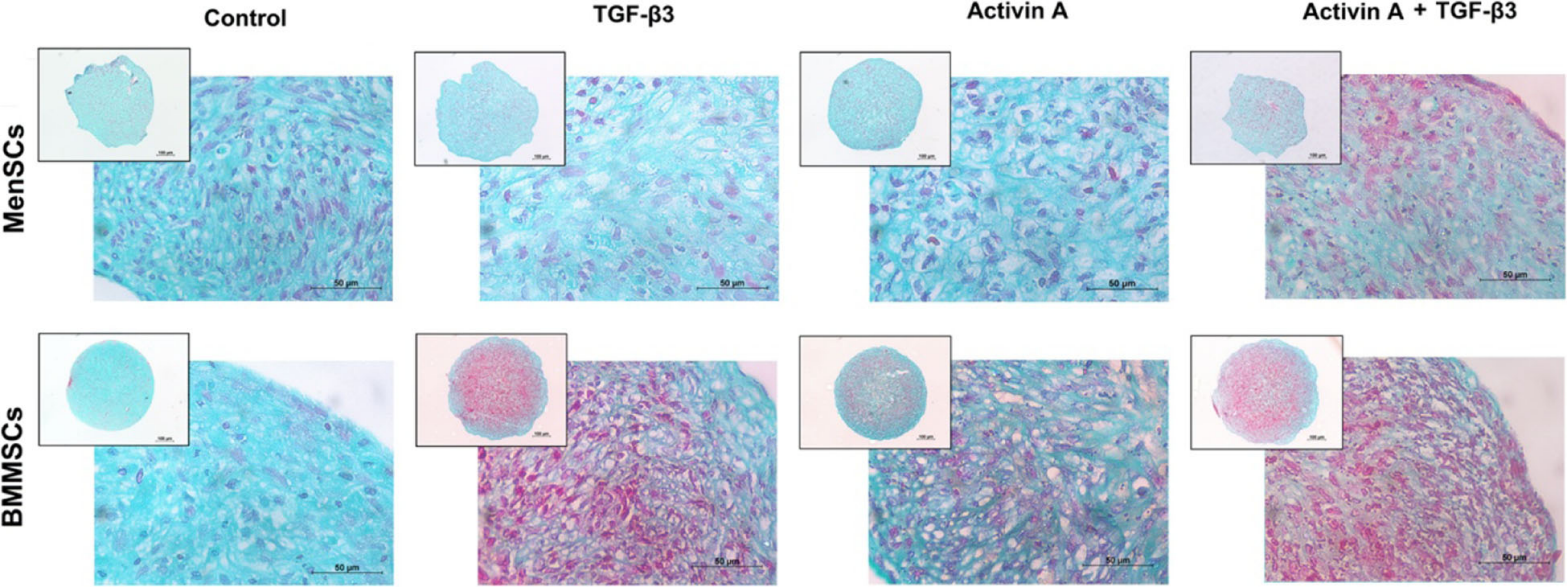
Chondrogenic differentiation of MenSCs and BMMSCs stimulated with activin A (50 ng/mL), TGF-β3 (10 ng/mL), and both activin A and TGF-β3, where activin A was added only during first 2 days. Histological analysis of cell pellets (Safranin O staining after 21 days of chondrogenic stimulation). Control cells were cultivated in chondrogenic medium without growth factors. Scale bars 100 μm and 500 μm

According to histological staining of pellet sections with Safranin O, different responses to TGF-β3 and activin A were observed in MenSCs and BMMSCs (Fig. 6). Combination of activin A and TGF-β3 stimulated proteoglycan synthesis in MenSCs, while stimulation with either activin A or TGF-β3 alone had no clear effect on chondrogenic response in those cells. In BMMSCs, accumulation of proteoglycans was observed in both activin A- or TGF-β3-stimulated groups of BMMSCs; however, the effects of TGF-β3 were more pronounced.

**Fig. 7:**
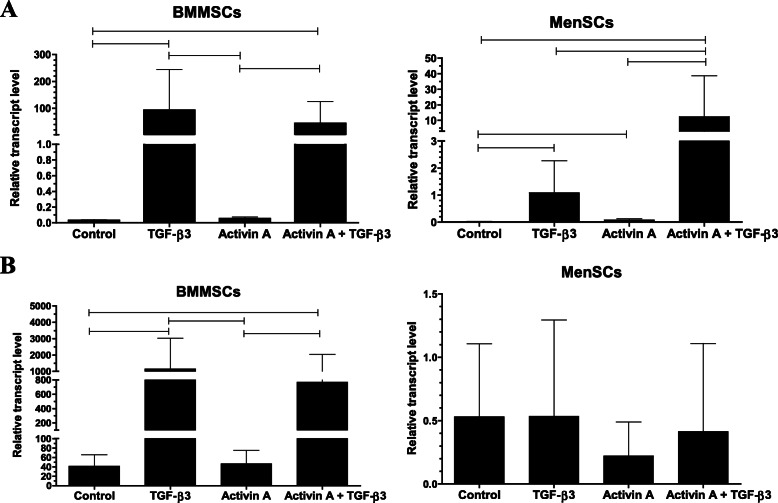
**a** Collagen type II (*COL2A1*) and **b** aggrecan (*ACAN*) gene expression in MenSCs and BMMSCs after stimulating chondrogenic differentiation for 21 days with activin A (50 ng/mL), TGF-β3 (10 ng/mL), and both TGF-β3 and activin A, where activin A was added only during first 2 days. Control cells were cultivated in the same chondrogenic medium without growth factors. Relative transcript level after normalization to geometric mean of B2M and RPS9 housekeeping genes. Data are presented as mean ± SD. Horizontal bars represent *p* ≤ 0.05

At gene expression level, a weaker ability to differentiate into chondrogenic lineage was observed in MenSCs, as compared to BMMSCs (Fig. 7a, b). Gene expression of collagen type II (COL2A1), the major structural and fibrillar collagen type in articular cartilage, was significantly lower in TGF-β3 and TGF-β3 with activin A groups of MenSCs, as compared to these two groups of BMMSCs (*p* ≤ 0.001). However, application of TGF-β3 alone or in combination with activin A significantly upregulated *COL2A1* expression in MenSCs, as compared to control, and the combination of activin A and TGF-β3 was significantly stronger than the effect of TGF-β3 alone, as compared to control (Fig. 7a). Activin A had no substantial effect on *COL2A1* gene expression in MenSCs.

Low levels of aggrecan (*ACAN)* gene expression were detected in all 4 conditions of MenSCs (Fig. 7b), as compared to substantially higher levels of its expression in BMMSCs. *ACAN* expression statistically significantly increased only in BMMSCs and only after stimulation with TGF-β3 or a combination of activin A with TGF-β3.

BMMSCs possessed a robust ability to differentiate into chondrogenic lineage with TGF-β3, according to *COL2A1* and *ACAN* gene expression (Fig. 7a, b). In opposite to MenSCs, combination of activin A with TGF-β3 in BMMSCs had no additional effect on the expression of the genes tested.

### Chondrogenesis of MenSCs and BMMSCs in vivo

Chondrogenesis studies in mice were performed as a classical in vivo model for analyzing the utility of cell loaded scaffolds for chondrogenesis, as discussed in [29].

**Fig. 8:**
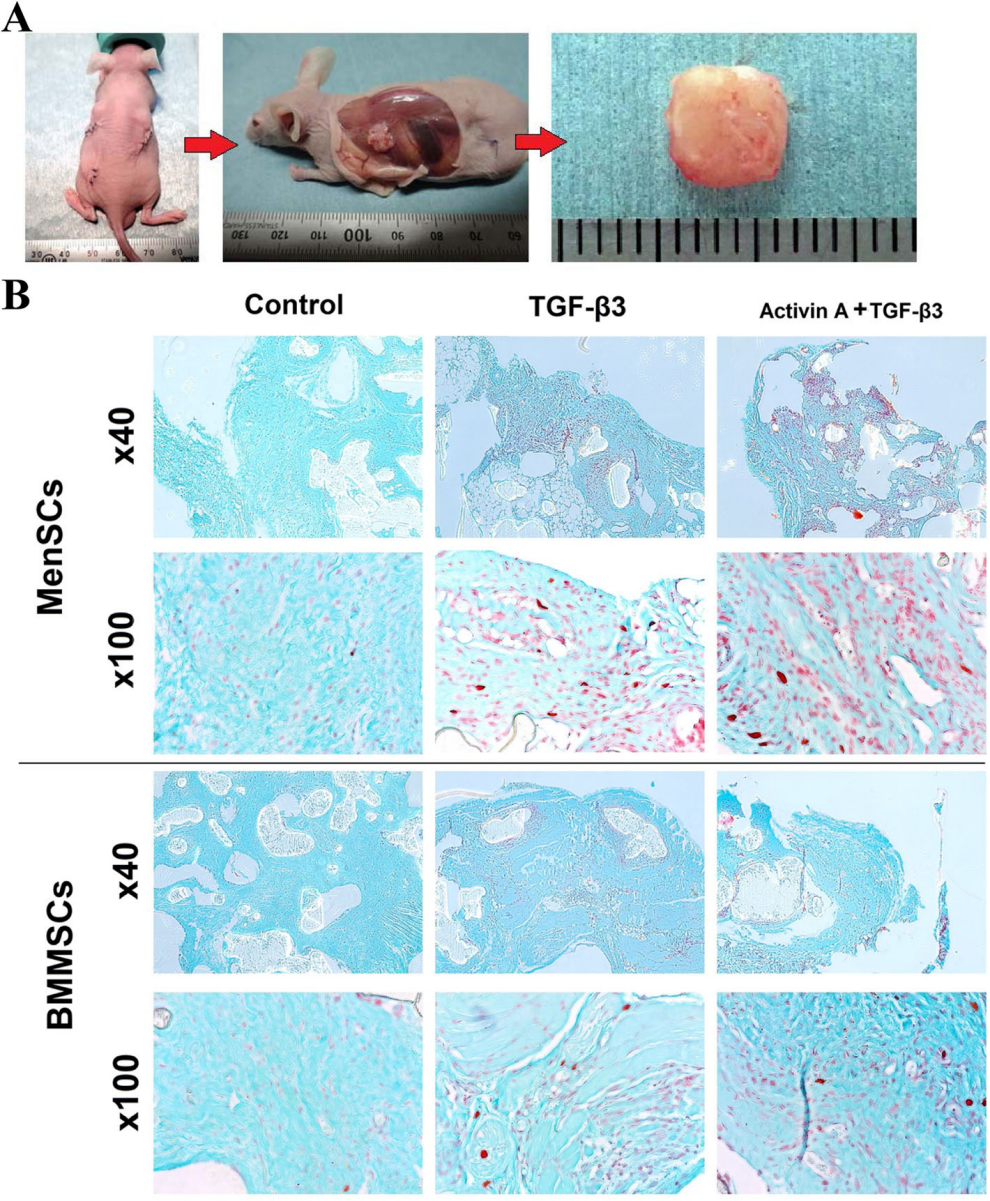
Atelocollagen/PLLA constructs with MenSCs and BMMSCs, stimulated by combination of activin A (50 ng/mL, first 2 days) and TGF-β3 (10 ng/mL, 21 days) or only with TGF-β3 (10 ng/mL, 21 days) or cultured without growth factors (21 days), followed by a subcutaneous insertion into mice for 9 weeks. **a** Experimental mouse with 3 cell-loaded constructs implanted subcutaneously; removal of implants at the end of experiment (9 weeks); removed construct. **b** Histological analysis of constructs after 9 weeks in vivo. Control constructs were differentiated under the same conditions, excluding growth factors. Safranin O staining. × 100 and × 400 magnification

According to Safranin O staining, higher accumulation of ECM proteins was observed in samples of combined activin A and TGF-β3 treatment, as compared to control in both cells (Fig. 8b).

**Fig. 9:**
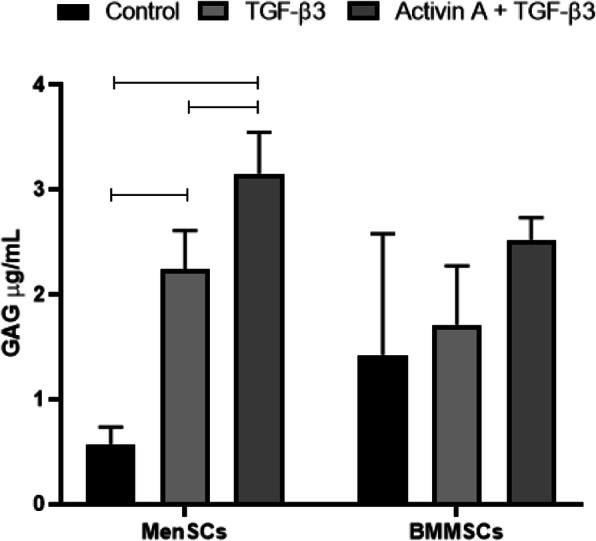
Production of GAGs in atelocollagen/PLLA constructs by MenSCs and BMMSCs, treated for 2 days with activin A (50 ng/mL), followed by 21 days with TGF-β3 (10 ng/mL), TGF-β3 (10 ng/mL), and subcutaneous insertion into mice for 9 weeks, spectrophotometrically measured by GAG assay. Control constructs were differentiated under the same conditions, but without growth factors. Data are presented as mean ± SD, horizontal bars represent *p* ≤ 0.05

Quantitative GAG synthesis was observed in samples treated with combination of TGF-β3 and activin A as well as single TGF-β3, in both MenSCs and BMMSCs (Fig. 9). However, statistically significant differences were observed only in MenSCs, where GAG production was higher in TGF-β3-stimulated group and additionally more pronounced if the combination of TGF-β3 + activin A was used. Although there were no significant differences between MenSC and BMMSC groups, MenSCs stimulated with TGF-β3 + activin A samples produced more GAGs, as compared to samples of BMMSCs under the same stimulation.

## Discussion

MSCs have been a focus in regenerative medicine due to their high potential to differentiate towards several lineages including osteoblasts, chondrocytes and adipocytes. Firstly, MSCs were discovered in bone marrow, while later, they were isolated from almost all human tissues, including the bone marrow, adipose tissue, umbilical cord blood, placenta [5, 6, 30–32]**,** and human menstrual blood [8, 33, 34]. The collection of menstrual blood samples does not require invasive procedures, which highlights a key advantage of MenSCs in regenerative medicine over BMMSCs [10]. MenSCs are increasingly investigated in different studies and are known to be similar to BMMSCs [8, 35, 36]. Furthermore, due to a high potential to differentiate into several different lineages, MenSCs have been proposed for treatment of critical limb ischemia, stroke, type I diabetes mellitus, Parkinson’s disease, and other neurodegenerative disorders in experimental disease models [9, 16]. Despite those advantages of MenSCs as compared to MSCs derived from other tissues, their characteristics have not been extensively studied so far. For instance, potential application of MenSCs for cartilage regeneration still requires additional studies on chondrogenic differentiation capacity. One of the usually used growth factors in chondrogenic differentiation of BMMSCs is TGF-β; however, other types of MSCs, for instance isolated from adipose tissue, may respond better to other growth factors, for instance BMP-2, BMP-4, or BMP-7 [37, 38]. The growth factor activin A is important in numerous cell processes, as well as the early stages of chondrogenesis [23]. Mechanisms of action of activin A and TGF-βs appear similar, they both induce phosphorylation of Smad2 and Smad3 during chondrogenesis [39]. However, the role of activin A in chondrogenic differentiation is controversial, as some studies propose that it antagonizes chondrogenesis and suppresses the expression of chondrogenic genes [40, 41].

Furthermore, activin A is very important in regulating the female menstrual cycle and has various functions in reproduction [42–44], suggesting that it may modulate other functional characteristics of MenSCs, including differentiation.

In this study, MenSCs and BMMSCs were isolated and characterized and compared by MSC surface marker expression (flow cytometry), proliferation abilities (CCK-8, spectrophotometry), and MSC trilineage differentiation capacity (osteogenic, adipogenic, chondrogenic). We observed higher proliferation and migration in MenSCs compared to BMMSCs, which is similar to the results obtained in other studies, where MenSC and BMMSC proliferation was investigated [35, 45].

Expression of typical MSCs surface markers (CD44, CD73, CD90, CD105), hematopoietic stem cell markers (CD14 CD34, CD36, CD45), and pluripotent stem cell markers (CD10, CD54, CD55, CD63, Notch1) were analyzed for characterization of MenSCs phenotype [46–50].

Expression of CD55, CD54, CD10, and Notch1 surface markers in MenSCs was significantly higher, as compared to BMMSCs, whereas expression of CD63 was higher in BMMSCs (Fig. 1). CD10 is a membrane metallo-endopeptidase, also known as neprilysin, which is involved in different signaling pathways influencing cell migration, angiogenesis, tumorigenesis, and immunomodulation [46]. Moreover, CD10 has also been a marker of highly proliferating cells, as well as calcifying cells [51], which was also observed for MenSCs in the present study. Higher expression of CD63 in BMMSCs has been associated with redifferentiation of chondrocytes in culture, which might reflect higher TGF-B induced chondrogenic differentiation potential observed in our study [52].

CD55 is a complement decay-accelerating factor, also a MSC marker used to identify endoderm in early embryonic development [53]. CD54 is an intercellular adhesion molecule-1 (ICAM-1), which was shown to be involved in the osteogenic and adipogenic differentiation of MSCs [54]. Noteworthy, we have previously demonstrated significantly stronger expression of CD54 and CD55 markers in ALDH positive chondrocytes which is associated with chondrogenic, Sox9, and Col type II positive phenotype [55]. In addition, higher expression of CD55 and CD54 in MenSCs may appear an advantageous immunosuppressive feature, as CD54 has been shown to interact with pro-inflammatory macrophages and increase immunosuppressive functions in MSCs [56].

Notch1 is a member of the Notch family of receptors and plays a significant role in mediating BMP9-induced osteogenic differentiation in MSC [57]. Notch is also known to be associated with enhanced MSC proliferation [58], which might be associated with high regenerative abilities of endometrium and could represent an advantage in regenerative medicine when large amounts of cells are needed.

Even though the main focus of this study was chondrogenic differentiation in both MenSCs and BMMSCs, we also compared their potential to differentiate into adipogenic and osteogenic lineages. MenSCs showed weak adipogenic differentiation capacity, which is in agreement to the data obtained in other studies [35, 45]. Moreover, the weak adipogenic differentiation capacity was also observed in amniotic fluid, placenta, umbilical cord, and umbilical cord blood MSCs [59, 60], which are of similar origin with MenSCs. This is in contrast to classical trilineage differentiation of BMMSCs. However, as described above, potential of MenSC to differentiate into neurogenic, cardiomyogenic, and even hepatogenic cell lineages [13–15] confirm their advanced differentiation potential, while the weaker adipogenesis probably indicate different differentiation profile rather than lack of stem cell properties. Furthermore, osteogenic differentiation capacity of MenSCs was significantly stronger as compared to BMMSCs (Fig. 2), which, to the best of our knowledge, has not been demonstrated previously. Stronger osteogenic differentiation capacity than in BMMSCs has been already reported for MSCs derived from placental and amniotic fluid [61–63], which are the ones of the closest origin to MenSCs, therefore, might share some functional similarities.

The levels of secreted activin A were measured during cell proliferation in monolayer and their chondrogenic differentiation. Higher levels of activin A were secreted by MenSCs in monolayer (Fig. 4), as compared to BMMSCs, and the elevated levels remained until 3rd day of chondrogenic differentiation in both non-stimulated control and TGF-β3 group of MenSC (Fig. 5). No significant chondrogenic response to activin A has been previously reported in BMMSCs [40, 41], which is in agreement with results obtained in this study. However, this is the first report of MenSCs stimulated to differentiate into chondrogenic lineage using activin A. Noteworthy, the expression of chondrogenic genes indicates weaker ability MenSCs to differentiate into chondrogenic lineage, in comparison to the levels in BMMSCs.

The response to activin A during chondrogenic differentiation was also different in MenSCs and BMMSCs. In MenSCs, the effects of activin A alone were negligible and only in combination with TGF-β3 resulted in a substantial increase of *COL2A1* expression. Even though *COL2A1* and *ACAN* gene expression was stronger in BMMSCs after stimulation with TGF-β3, activin A had no significant effect on the expression of these genes neither alone nor in combination with TGF-β3. Positive Safranin O staining was observed in histological samples of activin A-treated BMMSCs pellets, suggesting stimulatory effects of this factor on chondrogenic differentiation. In BMMSCs stimulated with a combination of activin A with TGF-β3, the expression of *COL2A1* gene (Fig. 7a) as well as the ECM protein content observed in histology samples (Fig. 6) were not significantly different, as compared to a single TGF-β3 stimulation.

The in vitro chondrogenesis studies using activin A were followed by an in vivo study. The technology of atelocollagen/PLLA construct application was chosen due to its biocompatibility and efficacy for stimulation of BMMSCs chondrogenic differentiation in vivo [29, 64]. Chondrogenesis of MenSCs and BMMSCs in constructs was induced for 3 weeks in vitro, followed by their subcutaneous insertion into nude BALB/C mice. Histological evaluation of the samples (Fig. 8), as well as GAG analysis (Fig. 9), indicated that the additional stimulation with activin A, prior to switching to TGF-β3, upregulated the production of ECM proteins in MenSCs, as compared to the controls or TGF-β3 stimulated samples. Somewhat similar tendencies, although less expressed, were observed in BMMSCs. These data are in contrast to the results obtained during in vitro cultures, where potential of MenSCs to differentiate into chondrogenic lineage was lower, as compared to BMMSCs. It is likely that longer stimulation is needed for MenSCs to effectively induce chondrogenic responses, as compared to BMMSCs, as in vivo study lasted an additional 63 days. Furthermore, paracrine effects in both cell types might also play a role in stimulating host cell response and their migration, which induces differentiation of MSCs [65]. MenSCs produce some unique factors, as demonstrated by others and in the present study, and seem less responsive to cytokine activation and express less immunosuppressive molecules, as compared to BMMSCs [10]. The differences in secretion of various paracrine factors might result in the disparities of host cell responses to MenSCs and BMMSCs in vivo*,* which in turn led to more expressed chondrogenic induction. It should also be considered that application of the scaffolds may play a role in stimulation of ECM production in MSCs, as previously demonstrated [17, 66]. Although it might appear that collagen-based scaffolds are essential for stimulating chondrogenesis in MenSCs, further studies are needed to support their use. Moreover, there are many soluble factors in mouse body fluids that may differently foster chondrogenic differentiation of implanted cells. Taken together, combined in vitro and in vivo data suggest that MenSCs differently respond to external stimuli and show distinct functional activity from BMMSCs. Nevertheless, it is likely that they are capable of switching to chondrogenesis, but this process takes longer time and the choice of suitable combinations of the factors to guide these cells and orchestrate the differentiation process are needed.

## Conclusions

The results presented in this original investigation demonstrate that MenSCs share many similar phenotypic properties with BMMSCs, but they exhibit several differences as well. MenSCs possess higher proliferation rate and osteogenic differentiation capacity than of BMMSCs, while adipogenic differentiation capacity is lower, which implies potential advantage for MenSCs for skeletal applications. Differences in stem cell surface markers and secreted levels of activin A indicate unique functions of MenSCs, where activin A seems to be more involved, as compared to BMMSCs. The potential of BMMSCs to differentiate into the chondrogenic lineage was more pronounced under stimulation with TGF-β3 in vitro. In contrast, the efficacy of activin A was higher in chondrogenic differentiation of MenSCs, as compared to BMMSCs, both in vivo and, particularly, in vitro. These data suggest that activin A is differently involved in the induction of chondrogenic differentiation in MenSCs vs. BMMSCs, which implies distinct pathways of chondrogenesis regulation in these cells. Following further optimization of the differentiation protocol and growth factor choice, potentially including activin A and extending the duration of growth factor stimulation, MenSCs may indeed turn out to be a promising population of stem cells for the further development as a key component of cell-based therapies for stimulating cartilage repair and regeneration.

## Data Availability

All relevant data are included in the article and/or its supplementary information files.

## Abbreviations

ACAN: Aggrecan
BMMSCs: Bone marrow MSCs
BMP-2: Bone morphogenetic protein-2
CCK-8: Cell counting kit 8
COL2A1: Collagen type II alpha 1 chain gene
DMEM: Dulbecco’s modified Eagle medium
ECM: Extracellular matrix
FGF-8: Fibroblast growth factor 8
GAG: Glycosaminoglycan
MenSCs: Menstrual blood-derived MSCs
MSCs: Mesenchymal stem cells
OA: Osteoarthritis
PLLA: Polylactic acid
TGF-β: Transforming growth factor β
